# Inter- and Intraobserver Variability in Bowel Preparation Scoring for Colon Capsule Endoscopy: Impact of AI-Assisted Assessment Feasibility Study

**DOI:** 10.3390/cancers17172840

**Published:** 2025-08-29

**Authors:** Ian Io Lei, Daniel R. Gaya, Alexander Robertson, Benedicte Schelde-Olesen, Alice Mapiye, Anirudh Bhandare, Bei Bei Lui, Chander Shekhar, Ursula Valentiner, Pere Gilabert, Pablo Laiz, Santi Segui, Nicholas Parsons, Cristiana Huhulea, Hagen Wenzek, Elizabeth White, Anastasios Koulaouzidis, Ramesh P. Arasaradnam

**Affiliations:** 1 Institute of Precision Diagnostics & Translational Medicine, University Hospital of Coventry and Warwickshire, Clifford Bridge Rd, Coventry CV2 2DX, UK; beibei.liu@uhcw.nhs.uk (B.B.L.); chander.shekhar@uhcw.nhs.uk (C.S.); cristiana.huhulea@uhcw.nhs.uk (C.H.); r.arasaradnam@warwick.ac.uk (R.P.A.); 2 School of Medicine, University of Warwick, Coventry CV4 7AL, UK; 3 Department of Gastroenterology, Glasgow Royal Infirmary, Glasgow G4 0SF, UK; daniel.gaya2@nhs.scot; 4 Department of Digestive Diseases, University Hospitals of Leicester NHS Trust, Leicester LE1 7RH, UK; alexander.robertson7@nhs.net (A.R.); alice.mapiye@uhl-tr.nhs.uk (A.M.); 5 Surgical Research Unit, Odense University Hospital, 5700 Svendborg, Denmark; benedicte.schelde-olesen@rsyd.dk (B.S.-O.); akoulaouzidis@hotmail.com (A.K.); 6 Department of Gastroenterology, Royal Oldham Hospital, Northern Care Alliance, Rochdale Road, Oldham OL1 2JH, UK; anirudh.bhandare@nca.nhs.uk; 7 Institute of Anatomy and Experimental Morphology, University Medical Center Hamburg-Eppendorf, 20246 Hamburg, Germany; ursula@corphealth.de; 8 Mathematics and Computer Science Department, University of Barcelona, 58508007 Barcelona, Spain; pere.gilabert@ub.edu (P.G.); santi.segui@ub.edu (S.S.); 9 GI Digital, Inc., New York, NY 10017, USA; pablo@gi.digital (P.L.); hagen@gi.digital (H.W.); 10 Warwick Clinical Trials Unit, University of Warwick, Coventry CV4 7AL, UK; nick.parsons@warwick.ac.uk; 11 Corporate Health International, Inverness IV2 5NA, UK; liz@corphealth.co.uk; 12 Department of Gastroenterology, Pomeranian Medical University, 70-204 Szczecin, Poland; 13 Department of Surgery, OUH Svendborg Sygehus, 5700 Svendborg, Denmark; 14 Department of Clinical Research, University of Southern Denmark, 5230 Odense, Denmark; 15 Leicester Cancer Centre, University of Leicester, Leicester LE1 7RH, UK

**Keywords:** artificial intelligence, machine learning, colon capsule endoscopy, panenteric capsule endoscopy, colonoscopy, bowel preparation, interobserver agreement, intraobserver agreement, Leighton–Rex grading scale, Colon Capsule CLEansing Assessment and Report, TransUNet segmentation model, reading workflow

## Abstract

**Background:** Colon capsule endoscopy (CCE) has seen increased adoption since the COVID-19 pandemic, offering a non-invasive alternative for lower gastrointestinal investigations. However, inadequate bowel preparation remains a key limitation, often leading to higher conversion rates to colonoscopy. Manual assessment of bowel cleanliness is inherently subjective and marked by high interobserver variability. Recent advances in artificial intelligence (AI) have enabled automated cleansing scores that not only standardise assessment and reduce variability but also align with the emerging semi-automated AI reading workflow, which highlights only clinically significant frames. As full video review becomes less routine, reliable, and consistent, cleansing evaluation is essential, positioning bowel preparation AI as a critical enabler of diagnostic accuracy and scalable CCE deployment. **Objective:** This CESCAIL sub-study aimed to (1) evaluate interobserver agreement in CCE bowel cleansing assessment using two established scoring systems, and (2) determine the impact of AI-assisted scoring, specifically a TransUNet-based segmentation model with a custom Patch Loss function, on both interobserver and intraobserver agreement compared to manual assessment. **Methods:** As part of the CESCAIL study, twenty-five CCE videos were randomly selected from 673 participants. Nine readers with varying CCE experience scored bowel cleanliness using the Leighton–Rex and CC-CLEAR scales. After a minimum 8-week washout, the same readers reassessed the videos using AI-assisted CC-CLEAR scores. Interobserver variability was evaluated using bootstrapped intraclass correlation coefficients (ICC) and Fleiss’ Kappa; intraobserver variability was assessed with weighted Cohen’s Kappa, paired t-tests, and Two One-Sided Tests (TOSTs). **Results:** Leighton–Rex showed poor to fair agreement (Fleiss = 0.14; ICC = 0.55), while CC-CLEAR demonstrated fair to excellent agreement (Fleiss = 0.27; ICC = 0.90). AI-assisted CC-CLEAR achieved only moderate agreement overall (Fleiss = 0.27; ICC = 0.69), with weaker performance among less experienced readers (Fleiss = 0.15; ICC = 0.56). Intraobserver agreement was excellent (ICC > 0.75) for experienced readers but variable in others (ICC 0.03–0.80). AI-assisted scores were significantly lower than manual reads by 1.46 points (*p* < 0.001), potentially increasing conversion to colonoscopy. **Conclusions:** AI-assisted scoring did not improve interobserver agreement and may even reduce consistency amongst less experienced readers. The maintained agreement observed in experienced readers highlights its current value in experienced hands only. Further refinement, including spatial analysis integration, is needed for robust overall AI implementation in CCE.

## Simple Summary

This study assessed the reliability of AI-assisted bowel cleansing scoring in colon capsule endoscopy using the CC-CLEAR scale. While interobserver agreement was excellent with manual scoring among experienced readers, AI-assisted reads did not improve agreement but showed reduced consistency, particularly among less experienced users. The mean AI-assisted scores were significantly lower than manual scores, highlighting potential interpretive challenges. These findings suggest that AI’s effectiveness currently depends on user expertise, reinforcing the importance of further development and refinement required for a robust AI implementation in CCE.

## 1. Introduction

Colon capsule endoscopy (CCE) is a non-invasive method for assessing the mucosa of the colon with pan-enteric visualisation capabilities. Despite its potential, maintaining reproducibility and consistency of key measures such as bowel cleansing assessment remains difficult. Adequate bowel preparation is vital not only for improving mucosal visibility and polyp detection but also for correctly deciding if follow-up optical colonoscopy is necessary. According to current European Society of Gastrointestinal Endoscopy (ESGE) guidelines, insufficient cleansing requires further evaluation to confidently rule out pathology, especially polyps measuring ≥5 mm [1,2]. Although the Colon Capsule CLEansing Assessment and Report (CC-CLEAR) scale was developed as a more objective, quantitative tool [3], bowel cleansing assessment is naturally subjective [4,5]. Interobserver agreement varies, with several studies showing only moderate to good consensus, even among experienced CCE reviewers [6,7]. Some results further complicate interpretation, with conflicting evidence suggesting that the Leighton–Rex score may provide better interobserver agreement than CC-CLEAR [4].

The rise of artificial intelligence (AI) in capsule endoscopy has brought promising advances, especially in improving time efficiency [8]. For example, Spada et al. reported a nine-fold reduction in reading time for small bowel CE using AI-assisted systems [9], a finding confirmed by interim results from the Capsule Endoscopy at Scale through Enhanced AI Analysis (CESCAIL) study [10]. Most AI frameworks concentrate on extracting clinically relevant frames, allowing readers to skip large parts of unremarkable footage. However, this efficiency introduces a new limitation: by skipping through the video, readers are unable to thoroughly assess bowel cleansing quality, particularly for segmental scoring systems. Without a reliable AI model to evaluate bowel cleanliness across the entire CCE video, this change in workflow may undermine the rigour and reproducibility of cleansing assessments, potentially eroding confidence among clinicians and patients.

To support the semi-automated reading pathway, AI algorithms must evolve beyond polyp detection to provide contextual interpretation, including cleansing quality evaluation, pathology classification, and polyp matching, as highlighted by Esmaeil et al. [1]. While AI-assisted bowel cleansing scores have been proposed to reduce interobserver variability, prior studies primarily focused on frame-level analysis rather than video-level analysis [2,3]. These approaches fail to account for spatial and temporal continuity within colon segments, whereby cleansing should be judged across mucosal areas and over time rather than from isolated frames. In practice, areas initially poorly visualised might later be adequately assessed from a different angle or with capsule rocking, a factor not captured in frame-level scoring. A recent video-based study by Schelde-Olesen et al. further underscored the limitations of current AI models, demonstrating poor agreement between AI algorithms and human readers, likely due to variability in training data and subjective reference standards [4]. These findings suggest AI should be positioned as a supportive adjunct rather than a replacement for human assessment, an approach not previously explored in the literature. In addition, no study has examined intraobserver variability before and after AI-assisted bowel cleansing assessment, and the impact of such approaches on readers’ evaluations remains unknown. Table 1 summarises all AI-based bowel preparation studies in CCE identified in our literature review.”

**Figure 1:**
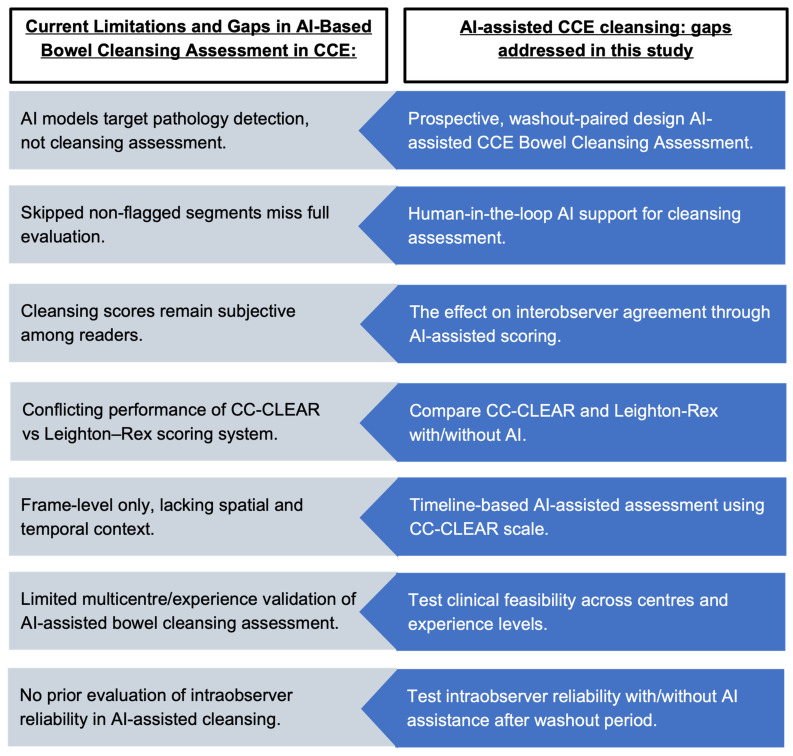
Summary of current gaps and study objectives for AI-assisted bowel cleansing in CCE.

Considering these challenges, our sub-study aimed to address these gaps by integrating an AI-assisted tool for objective bowel preparation scoring in CCE. The primary objective was to evaluate interobserver variability in bowel cleansing assessment within the standard reading arm, using both the Leighton–Rex and CC-CLEAR scoring systems, among readers with differing levels of experience in CCE. The secondary objective was to evaluate both the interobserver and intraobserver variability by comparing standard and AI-assisted readings of the same CCE videos among the same readers, following a washout period. Figure 1 summarises the current limitations in bowel cleansing assessment for CCE and outlines the objectives of this study in addressing those gaps. This prospective, multi-reader, washout-paired evaluation tests a hybrid human-in-the-loop AI-assisted bowel cleansing assessment tailored to CCE, rather than small bowel extrapolations, directly addressing standardisation and reproducibility at scale.

## 2. Methods

### 2.1. Study Design and Video Selection

In this study, 25 completed CCE videos, defined as those with capsule excretion before battery exhaustion, were pseudonymised and randomly selected from 673 videos in the CESCAIL multicenter prospective diagnostic accuracy study using the RAND function in Microsoft Excel (Microsoft Corporation, Redmond, WA, USA). Each video ID was assigned a random number, and the dataset was then sorted in ascending order based on these values. The first 25 entries were selected for inclusion [6]. The CESCAIL study investigated a Computer-Aided Detection (CADe) system for polyp detection in CCE using the PillCam™ COLON 2 system (Medtronic, Dublin, Ireland) [7]. The patient inclusion criteria were based on the NHS England pilot study, which included adults referred to secondary care under the urgent referral pathway for lower gastrointestinal (GI) symptoms [8] and those scheduled for post-polypectomy surveillance as part of their routine clinical care [9] (Supplementary Table S1 for the details of the inclusion criteria). The sole exclusion criterion for CESCAIL was the inability to provide informed consent.

A power analysis was conducted for both the paired comparative analysis and interobserver agreement. For the primary comparison between AI-assisted and clinician bowel cleanliness scores, a paired-sample design was assumed with an expected moderate effect size (Cohen’s d = 0.6), α = 0.05, and power = 80%. This yielded a required sample size of 24 paired observations; the current study includes 25, thus meeting power requirements. For the interobserver reliability analysis using the Intraclass Correlation Coefficient (ICC), we assumed a population ICC of 0.60, α = 0.05, 8 raters, and 25 subjects [10]. Using an F-distribution-based approximation [11], the calculated statistical power to detect an ICC of at least 0.60 was 1, sufficient for reliable ICC estimation.

### 2.2. CCE Readers: Grading of Bowel Cleansing 

**Figure 2:**
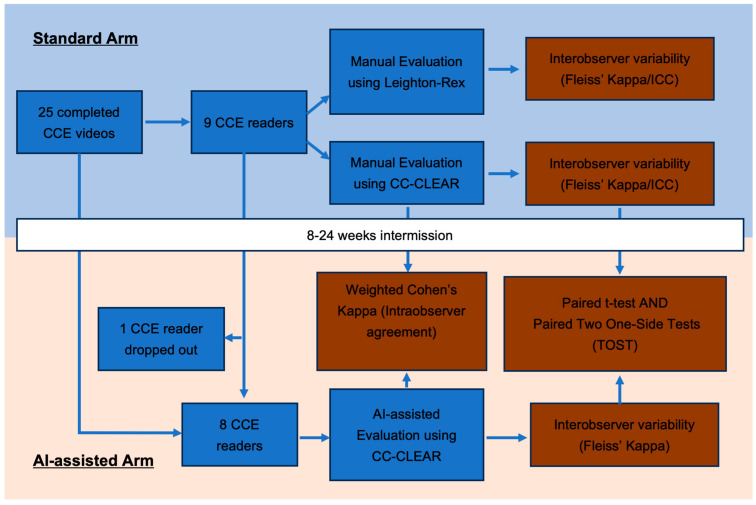
Study flowchart. CCE, colon capsule endoscopy.

This study employed two distinct video assessment arms: the standard and the AI-assisted reading arms (Figure 2). In the standard arm, accredited CCE readers with various experiences, ranging from 150 to 2000 cases, independently reviewed full-length videos at the maximum frame rate for bowel cleansing assessment only. An experienced reader is defined by more than 500 CCE lifetime reads in this study [12]. Readers’ experiences are detailed in Supplementary Tables S2 and S3. Key anatomical landmarks, including the first caecal image, hepatic flexure, splenic flexure, and final rectal image, were pre-marked by an expert reader to standardise assessments.

**Figure 3:**
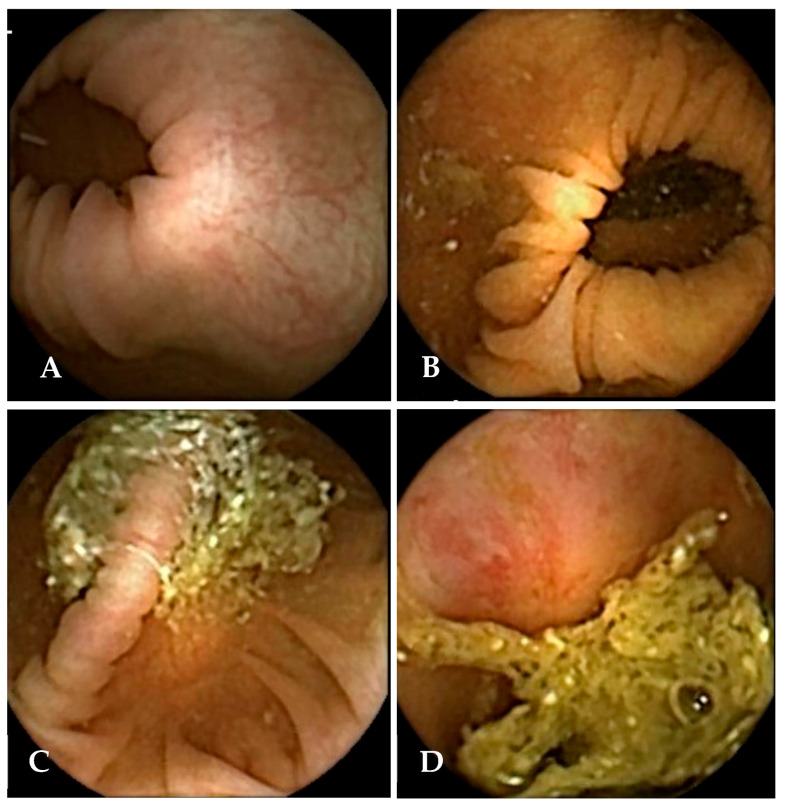
Examples of colon capsule endoscopy frames graded according to the Leighton–Rex scale: (**A**) excellent, (**B**) good, (**C**) fair, and (**D**) poor.

During the review, readers evaluated bowel cleansing quality using both the Leighton–Rex [13] and CC-CLEAR [14] scoring systems. The Leighton–Rex scale was applied using a 4-point score (poor, fair, good, excellent), in which only “fair,” “good,” and “excellent” were considered adequate (Figure 3). For an examination to be considered overall adequate on this scale, all five colonic segments had to meet the threshold for acceptable cleansing. In contrast, the CC-CLEAR scale employs a more quantitative approach across three colonic segments: the right colon, the transverse colon, and the left colon. Within each segment, cleansing is scored from 0 to 3 points based on the percentage of mucosa visualised (<50%  =  0 points, 50–75%  =  1 point, >75%  =  2 points, and >90%  =  3 points). The total score, obtained by summing the segment scores, categorises overall bowel cleanliness as excellent (8–9), good (6–7), or inadequate (0–5).

### 2.3. AI-Assisted Cleansing Grading

**Figure 4:**
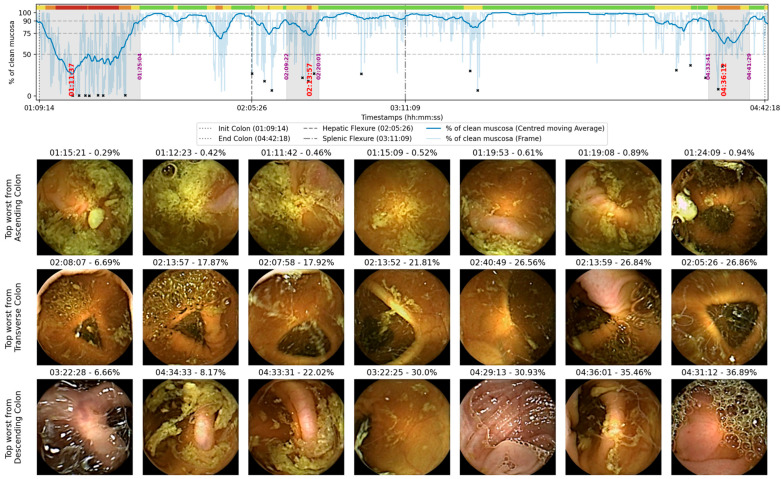
Example of an AI-generated output displaying the cleansing timeline, colour-coded according to CC-CLEAR scores. The timeline shows the percentage of mucosal cleanliness over time: green (>90%), yellow (75–90%), orange (50–75%), and red (<50%). Red timestamps indicate the worst image within each colonic segment, while dotted black lines mark the colonic flexures. The grey zones indicate time intervals containing the worst images, corresponding to segments where the cleanliness graph falls below 90% and subsequently rises above 90%, with the start and end points marked by purple timestamps. The images at the bottom represent the seven worst frames selected by the AI for each segment.

The AI algorithm used in this sub-study was developed by our collaborators, Gilabert et al., to support clinicians in evaluating bowel cleanliness in CCE using the CC-CLEAR scale. The system combines image segmentation and classification to estimate mucosal visibility across the entire video while significantly reducing CCE experts’ annotation burden during its training phase. It employs a TransUNet architecture trained to detect intraluminal content in capsule frames, guided by a custom “Patch Loss” function that relies on binary patch-level labels “clean” or “dirty”, rather than full-frame manual segmentation [15,16]. During model development, the following hyperparameters were tuned: (i) patch size for segmentation; (ii) Gaussian smoothing parameters; (iii) TransUNet architecture settings (depth/heads); and (iv) learning rate. Cleanliness is calculated on a frame-by-frame basis by quantifying the proportion of visible mucosa. This information is then summarised in a timeline plot, illustrating fluctuations in bowel cleanliness throughout the capsule examination journey. From this continuous analysis, the algorithm extracts features aligned with CC-CLEAR thresholds and classifies video segments into corresponding cleanliness categories (scores 0–3). Per-frame visible-mucosa proportion was mapped deterministically to CC-CLEAR thresholds: <50% = 0 points, 50–75% = 1 point, 75–90% = 2 points, and >90% = 3 points; these cut-offs were not learned by the model but applied to its per-frame predictions. This system is designed to enhance reader efficiency while preserving clinical control, supporting a reader-led, AI-assisted workflow (Figure 4 for an example of the AI output). A detailed description of the algorithm’s training, validation, and optimisation can be found in the work by Gilabert et al. [15,16,17]. The model was trained, validated, and tested on 113 videos (69/22/22), with splitting performed at the patient level to prevent data leakage.

In addition to generating a timeline plot, the algorithm identifies and flags the six lowest bowel cleansing quality frames within each colonic segment, providing corresponding timestamps. These frames are selected according to the lowest predicted mucosal visibility, without independent validation of this approach. This fixed number was selected to optimise clinical usability by fitting clearly into a single-page report format, allowing high-resolution image display without cognitive overload. The approach follows a “worst-first” principle, whereby if the most poorly visualised frames in a colonic segment are deemed adequate, the remainder of the segment can reasonably be assumed adequate. Conversely, if the worst frames or sections are inadequate, the whole segment would be considered inadequate overall, prompting a follow-up colonoscopy regardless of the remainder. To maintain reader autonomy, flagged frames were accompanied by timestamps, allowing further review of adjacent video segments when needed. This strategy supports a semi-automated, human-in-the-loop workflow and represents a practical first step in validating AI-assisted cleansing evaluation in clinical settings.

Extending on the work of Gilabert et al., our study required all original readers from the initial standard read to undergo an 8–24-week washout period to minimise recall and reporting bias before reassessing the same 25 videos in the AI-assisted arm. Readers were briefed on the AI-assisted reading approach using a detailed instruction document, and optional supplementary training was provided either in person or via virtual meetings to ensure consistency in interpretation. During this phase, readers were limited to the AI-generated visual guide, which included six flagged frames per segment along with the option to review a small number of adjacent frames via RAPID software v9 [18] as needed. As the AI output was based on the CC-CLEAR scale, assessments in the AI-assisted arm were limited to CC-CLEAR scoring only (Figure 3). To minimise bias, all readers were blinded to each other’s scores during both rounds.

All datasets were assessed for missing values before statistical analysis. If missing data were present, the pattern and extent of missingness were examined. Given the observational design, we planned to exclude data points with missing values if they were minimal, non-systematic, and unlikely to bias the results. No imputation was planned unless missingness exceeded 5% or showed a systematic pattern [19]. For interobserver agreement analyses, any missing reader scores were omitted on a per-segment basis.

### 2.4. Statistical Analysis

Interobserver agreement among CCE readers, with and without AI assistance, was assessed using Fleiss’ Kappa, with bootstrapping (1000 iterations) applied to estimate 95% confidence intervals. Fleiss’s equally arbitrary guidelines characterise Kappas over 0.75 as excellent, 0.40 to 0.75 as fair to good, and below 0.40 as poor [20]. Agreement was evaluated, including overall and by colonic segment, using both the Leighton–Rex and CC-CLEAR scoring systems. Although intraclass correlation coefficients (ICC) have limitations when applied to categorical data, it was included in this study to maintain consistency with previous literature, where it has been commonly used to evaluate the overall reliability across raters in bowel cleansing assessment [10,21,22]. Given that the scoring systems used in CCE represent quasi-continuous ordinal scales, ICC was used alongside Fleiss’ kappa to enhance comparability with previous work and to offer a comprehensive picture of interobserver variability. Agreement levels were interpreted using the criteria established by Landis and Koch, which classify values < 0 as no agreement, 0–0.20 as slight, 0.21–0.40 as fair, 0.41–0.60 as moderate, 0.61–0.80 as substantial, and 0.81–1.00 as almost perfect agreement [23].

Intraobserver variability comparing the agreement of standard and AI-assisted reads by the same reader was assessed using weighted Cohen’s Kappa (κ) to account for the ordinal nature of the CC-CLEAR scores. To evaluate whether AI-assisted readings were clinically equivalent to standard clinician readings, both the paired t-test and Two One-Sided Tests (TOST) methodologies were applied [24]. Equivalence bounds were defined as ±1 CC-CLEAR point, representing the maximum difference considered clinically acceptable. For the paired TOST, equivalence was concluded if both one-sided tests yielded statistically significant results (*p* < 0.05). All statistical analyses were conducted in R version 2025.05.1 [25] using the following packages: “psych” for reliability analysis [26], “dplyr” [26] for data manipulation, “effsize” [27] for effect size analysis, “irr” [28] for agreement measures, “boot” for bootstrapped confidence intervals [29], and “TOSTER” for equivalence testing. All visualisations were created using the “ggplot2” package [26].

To assess the stability of interobserver agreement in both the manual and AI-assisted reads, a sensitivity analysis was performed using a leave-one-observer-out approach. This method systematically excludes one observer at a time to determine whether any individual rater disproportionately influences the overall agreement. This was important to address in the study design, in which one observer dropped out after the washout period, potentially impacting the reliability of consensus. Agreement was quantified using both Fleiss’ Kappa and ICC. For each reduced set of observers, we computed the agreement statistics and used a non-parametric bootstrap procedure with 1000 replicates to estimate 95% confidence intervals. The Bias-Corrected and Accelerated (BCa) method was employed via the “boot.ci” function (type = “bca”) from the R “boot” package. To evaluate whether the agreement values obtained after excluding an observer differed significantly from the overall mean, empirical two-tailed *p*-values were calculated from the bootstrap distribution.

### 2.5. Ethical Approval and Funding

The CESCAIL study received ethical approval from the Southwest–Central Bristol Research Ethics Committee (REC reference: 21/SW/0169) and was registered on ClinicalTrials.gov (NCT06008847). The main study was funded by the National Institute for Health and Care Research (NIHR) through the AI Award programme (Award number: NIHR AI_AWARD02440). The design, conduct, data collection, analysis, and reporting of this study were carried out independently of the funders. All participants provided written informed consent after receiving verbal and written information about this study.

## 3. Results

The evaluations from both the standard and AI-assisted bowel cleansing assessments of 25 videos, including interobserver and intraobserver agreements, are summarised in Table 2 and Table 3. One reader dropped out following an extended 6-month period of intermission. For the Leighton–Rex scores, interobserver agreement was poor, with a Fleiss’ Kappa of 0.15, and moderate agreement on the ICC of 0.55. In contrast, the CC-CLEAR score showed fair agreement, as indicated by Fleiss’ Kappa of 0.27, and excellent agreement by ICC (0.90). Subgroup analyses revealed that experienced readers demonstrated marginally higher agreement than less experienced readers in both scoring systems (Table 2).

In the AI-assisted arm, the agreement did not consistently improve. When accounting for sampling variability via bootstrap resampling (appropriate given the smaller sample size), Fleiss’ Kappa decreased to 0.14 vs. 0.27 for CC-CLEAR, and bootstrapped ICCs were also reduced to 0.59 vs. 0.69 for CC-CLEAR. Subgroup analysis indicated that experienced readers maintained higher interobserver agreement (Fleiss’ Kappa: 0.41, ICC: 0.87) compared to less experienced readers (Fleiss’ Kappa: 0.15, ICC: 0.56). When comparing interobserver agreement between the standard and AI-assisted arms, bootstrapped ICC values were consistently lower in the AI-assisted read compared to the standard read (Table 2). A paired *t*-test of raw CC-CLEAR scores showed a mean difference of −1.46 points (95% CI: −1.58 to −1.33; *p* < 0.001) in the AI-assisted read when compared to the standard read, supported by a Cohen’s d of –0.74 (indicating a moderate-to-large effect size) (see Table 3). TOST analyses further confirmed statistically significant differences in scoring between AI-assisted and standard reads across all readers, consistent with the decline in CC-CLEAR scores observed in the paired *t*-test in the AI-assisted read (Supplementary Table S4). These findings suggest that AI-assisted scoring did not enhance interobserver agreement and may have reduced scoring consistency, particularly among less experienced readers.

Intraobserver agreement, assessed by comparing each reader’s standard and AI-assisted scores, was excellent among all experienced readers. In contrast, half of the less experienced readers demonstrated poor or no agreement. These patterns were consistent across both ICC and weighted Cohen’s Kappa (κ) metrics (Table 3).

The sensitivity analysis revealed no statistically significant outliers in the manual read for either ICC or Fleiss’ Kappa, nor in the AI-assisted ICC (see Supplementary Tables S5–S7 and Figure S1). However, the AI-assisted Fleiss’ Kappa analysis identified four observers whose exclusion led to statistically significant changes in agreement (*p* < 0.05) in Table S8 and Figure S2. Notably, the removal of two less experienced observers from the same centre resulted in a notable increase in agreement. In contrast, the exclusion of two other observers, one experienced and one inexperienced, both from a different centre, led to a decrease in agreement. These findings suggested the influence of individual raters and institutional contexts on interobserver reliability within AI-assisted evaluation frameworks only.

## 4. Discussion

In CCE, bowel preparation is traditionally assessed through full manual video review, conducted alongside the evaluation for colonic pathologies. However, as AI becomes increasingly embedded in clinical workflows, enhancing diagnostic efficiency, the necessity for traditional full CCE video review is anticipated to diminish. AI algorithms increasingly filter and prioritise the most relevant (typically positive for pathologies) frames, thereby reducing the time burden on readers. As a result, there is a growing need for efficient and reliable methods to assess bowel cleanliness without requiring full video examination. While several studies have evaluated AI-based bowel preparation assessment using manual readings as the reference standard. Most of them are image-based rather than video-based, potentially limiting their clinical applicability [2,3,30]. Notably, Schelde-Olesen et al. recently reported minimal agreement between AI output and CCE readers’ assessments when AI was used entirely autonomously on video-based analysis [4]. While high-quality reference standards may improve agreement between AI and human readers, excluding human oversight could undermine this agreement as well as the trustworthiness of AI-generated scores.

To our knowledge, this is the first study to address this issue by implementing an AI-assisted, rather than fully autonomous, bowel cleansing assessment, aligning with the principle of “keeping the human in the loop” [4]. Our evaluation centres on a human-in-the-loop workflow purpose-built for colon capsule, rather than fully autonomous, to test whether targeted AI guidance can standardise cleanliness scoring across readers with varying levels of experience. This hybrid approach was intended to preserve clinical control and judgement while improving workflow efficiency. Despite this, our findings revealed that interobserver agreement remained low, even with AI assistance. The interpretation of both the cleansing timeline and the selection of the six worst frames proved to remain highly subjective, particularly among less experienced readers. In subgroup analyses, experienced readers consistently demonstrated significantly higher agreement (Fleiss’ Kappa = 0.41, ICC = 0.87) compared to less experienced readers (Fleiss’ Kappa = 0.15, ICC = 0.56). This may be due to the experienced readers placing greater emphasis on visual assessment of the worst images, while less experienced readers tended to rely more heavily on the AI-computed cleansing scores displayed in the timeline (over-reliance from automation bias). Additionally, the AI algorithm was trained using annotations from expert CCE readers, rather than a mix of experience levels (miscalibration) [31]. This may partly explain the higher concordance observed among experienced readers and could also amplify reliance or create mismatches for novices. Future iterations should therefore include calibration across experience levels and integrate explicit user-feedback loops.

However, interpreting these timelines is complex and subject to several limitations. Firstly, unlike colonoscopy, the capsule’s bidirectional movement and dual-camera views allow for mucosal surfaces obscured in one frame to be visualised in another. The timeline’s per-frame cleansing estimates do not account for this spatiotemporal integration, which human readers often perform intuitively. A promising direction for future research is the integration of spatial mapping into AI algorithms, enabling them to recognise regions of the bowel that have been adequately visualised from multiple angles [1]. Such spatiotemporal modelling would not only enhance the accuracy of bowel cleansing assessment but would also be critical for reliable polyp localisation and for distinguishing multiple lesions within the same colonic segment [4,12]. This spatial localisation capability has already been used in the gastric magnetic capsule technologies [4].

Secondly, AI assessments are fully quantitative, based solely on the percentage of visible mucosa. In contrast, clinician assessments, even when using structured tools like the CC-CLEAR scale, retain a degree of subjectivity and qualitative interpretation. This discrepancy was evident in our intraobserver analysis, where most readers showed a statistically significant reduction in segment CC-CLEAR scores during AI-assisted assessments, as demonstrated by paired t-tests and TOST. On average, scores declined by 1.46 points on the CC-CLEAR scale in the AI-assisted read (Table 3 and Figure S3 in Supplementary Materials). Consequently, the lower AI-assisted cleansing scores may result in more patients being referred for unnecessary colonoscopy due to poor bowel cleansing, thereby affecting both cost-effectiveness and patient burden [4]. Importantly, although AI-assisted and manual reads were not statistically equivalent, this does not imply that the AI approach is inaccurate. Rather, the AI-assisted method failed to reproduce the outcomes of full manual assessment, particularly concerning clinical judgement and interobserver agreement. This limitation may also potentially stem from the “six worst frames” method, which, while conceptually sound, may not yet be the most optimised way to capture the true cleansing quality of a segment. Future studies should refine this approach, for instance, by selecting a larger or variable number of frames depending on segment quality, with more frames presented when cleansing is poor to provide a more accurate assessment. Currently, manual reader-based evaluation remains the reference standard, and AI tools will require further refinement to meet or surpass this benchmark before they can be adopted widely in clinical practice.

The sensitivity analysis revealed that readers who trained and worked closely together exhibited similar interpretive patterns. Notably, the removal of two readers from the same institution, one of whom was a nurse routinely pre-reading for a consultant, led to a decrease in overall agreement, while the removal of another reader pair from a different centre with a similar nurse–consultant dynamic increased agreement. These findings suggest that institutional training environments and shared interpretive frameworks can significantly shape scoring behaviour and influence interobserver reliability. Despite the subjective nature of bowel preparation assessment, the results demonstrated the potential for harmonised training to enhance consistency, particularly in AI-assisted workflows. This further reinforces the necessity of external validation through multicentre studies to ensure the generalisability of AI-assisted approaches.

Moreover, our results reaffirm prior studies indicating that the CC-CLEAR score yields higher interobserver consistency compared to Leighton–Rex. In our study, CC-CLEAR showed better agreement (Fleiss’ Kappa = 0.27, ICC = 0.90) than Leighton–Rex (Fleiss’ Kappa = 0.15, ICC = 0.55), consistent with prior literature [14].

Finally, a major limitation of this study is the small number of readers, with the dropout of one experienced reader potentially introducing bias. Another limitation of this study is the lack of direct evaluation and comparison of reading efficiency between the two arms. The potential efficiency gain remains theoretical and requires validation in prospective time-and-motion studies. In this assisted paradigm, a potential clinical risk is that conservatively low AI-generated cleansing scores could trigger unnecessary conversion to colonoscopy when a segment might otherwise be judged adequate on full review. While AI-assisted reading of CCE is feasible, further refinement is essential to improve intraobserver and interobserver agreements and foster greater trust among clinicians. Future studies should involve larger and more diverse reader cohorts, ideally incorporating a qualitative component to explore the dynamics of reader–AI interaction. Understanding the human factors that shape trust, reliance, and interpretation of AI-generated outputs will be critical to the effective and sustainable integration of AI into clinical CCE workflows.

## 5. Conclusions

In summary, AI assistance did not improve interobserver agreement overall and, in fact, reduced consistency among less-experienced readers, whereas experienced readers maintained excellent intraobserver reliability. These findings highlight that the effectiveness of AI-assisted interpretation remains highly dependent on reader experience. Future study should prioritise spatial and segmental mapping, as well as user-level calibration, to improve accuracy of bowel cleansing assessment, prevent unnecessary colonoscopy conversions, and support standardised adoption across centres. Importantly, cleansing evaluation represents only one element of the broader algorithmic framework needed to deliver a fully integrated AI-assisted CCE diagnostic service.

## Figures and Tables

**Table 1:** Summary of key studies evaluating AI-based bowel cleanliness assessment in CCE [5].

Study	Type of AI	Number of Videos/Frames Analysed	Level of Agreement AI with Readers, %	Sensitivity	Specificity
Buijs [2]	Non-linear index model SVM mode	41 videos 41 videos	32% 47%	- -	- -
Becq [3]	R/G ratio R/(R + G) ratio	216 frames 192 frames	- -	86.5% 95.5%	77.7% 62.9%
Schelde-Olesen [4]	Pixel-level classification was performed using models as originally described by Buijs et al. [2]	842 videos	Cohen’s k = 0.02–0.17 on the 2-point scale Cohen’s k = 0.02–0.16 on the 4-point scale	-	-

**Table 2:** Summary of the interobserver agreement of both standard read and AI-assisted arms.

Interobserver Agreement–Standard Read				
Readers (*n* = 9)	Fleiss Kappa	Boostrapped Fleiss Kappa (95%CI)	ICC	Bootstrapped ICC (95%CI)
Leighton–Rex (all)	0.15	0.15 (0.11–0.18)	0.55	0.55 (0.48–0.62)
Experienced readers	0.18	0.18 (0.13–0.24)	0.60	0.60 (0.54–0.67)
Less experienced readers	0.12	0.12 (0.06–0.18)	0.54	0.53 (0.46–0.63)
CC-Clear (all)	0.27	0.27 (0.23–0.30)	0.90	0.90 (0.86–0.92)
Experienced readers	0.29	0.29 (0.24–0.25)	0.90	0.90 (0.87–0.92)
Less experienced readers	0.24	0.24 (0.18–0.29)	0.88	0.88 (0.83–0.91)
**Interobserver Agreement–AI-assisted Read**				
**Readers (*n* = 8)**	**Fleiss** **Kappa**	**Boostrapped Fleiss Kappa (95%CI)**	**ICC**	**Bootstrapped ICC (95%CI)**
CC-Clear (all)	0.27	0.14 (0.10–0.11)	0.69	0.59 (0.49–0.67)
Experienced readers	0.41	0.27 (0.21–0.33)	0.87	0.68 (0.60–0.75)
Less experienced readers	0.15	−0.034 (0.079–0.004)	0.56	0.51 (0.35–0.63)

**Table 3:** Intraobserver agreement within the same reader comparing standard vs. AI-assisted read using ICC and weighted Cohen’s Kappa.

Readers (*n* = 8)	CCE Readers	ICC (95%CI)	Weighted Cohen’s Kappa (*k*)	*p* Value
Experienced	Reader 1	0.77 (0.68–0.84)	0.316	<0.001
Experienced	Reader 2	0.90 (0.85–0.93)	0.321	<0.001
Experienced	Reader 3	0.81 (0.73–0.87)	0.338	<0.001
Experienced	Reader 4	0.78 (0.69–0.85)	0.352	<0.001
Less experienced	Reader 5	0.69 (0.57–0.78)	0.109	0.004
Less experienced	Reader 6	0.21 (0.02–0.39)	−0.007	0.771
Less experienced	Reader 7	0.03 (−0.16–0.23)	−0.031	0.178
Less experienced	Reader 8	0.80 (0.72–0.86)	−0.023	0.796
Comparing CC-CLEAR scores between standard and AI-assisted arms				
Paired *t*-test on Raw score	Mean difference = −1.46 (−1.58 to −1.33)		Cohen’s d (effect size) d = −0.74	*p* < 0.001

## Data Availability

Owing to GDPR restrictions, raw capsule videos cannot be shared. De-identified scoring matrices and complete R analysis scripts are available from the corresponding author upon reasonable request. Controlled on-site or secure-environment video access may be arranged subject to appropriate approvals.

## Supplementary Materials

The following supporting information can be downloaded at: https://www.mdpi.com/article/10.3390/cancers17172840/s1, Table S1: Inclusion and exclusion criteria for NHS England Criteria used in the CESCAIL study [4]. Table S2: The CCE Readers List. Table S3: CCE readers and experience. Table S4: Summary of Paired TSOT and NHST. Table S5: Sensitivity Analysis of Manual Reads Using Bootstrapped Fleiss’ Kappa. Table S6: Sensitivity Analysis of Manual Reads Using Bootstrapped ICC. Table S7: Sensitivity Analysis of AI-assisted Reads Using Bootstrapped ICC. Table S8. Sensitivity Analysis of AI-assisted Reads Using Bootstrapped Fleiss’ Kappa. Figure S1: Sensitivity analysis using a leave-one-observer-out approach: (a) Fleiss’ Kappa for manual reads; (b) Intraclass Correlation Coefficient (ICC) for manual reads. Figure S2: Sensitivity analysis using a leave-one-observer-out approach: (a) Fleiss’ Kappa values after sequential removal of each observer. Removal of AM and AR resulted in lower overall agreement, while removal of BBL and CS improved agreement; (b) Intraclass Correlation Coefficient (ICC) for AI-assisted reads. Figure S3: Box plot to compare mean score in the AI-assisted arm against the standard arm using CC Clear score. The AI-assisted group shows consistently lower total CC-Clear scores compared to clinician-only ratings, indicating stricter or more conservative evaluations by the AI-assisted method. The boxes represent interquartile ranges (IQR), with horizontal lines indicating medians, and whiskers denoting 1.5× IQR.

## Abbreviations

AI	Artificial intelligence
AI-SPEED™	AI-assisted System for Polyp and Endoscopy Evaluation and Detection
BCa	Bias-Corrected and Accelerated (bootstrap method)
CADe	Computer-Aided Detection
CCE	Colon capsule endoscopy
CC-CLEAR	Colon Capsule CLEansing Assessment and Report
CE	Capsule endoscopy
CESCAIL	Capsule Endoscopy at Scale through Enhanced AI Analysis Study
CI (bootstrap)	Confidence interval from bootstrapping
Fleiss’ Kappa	Statistical measure for multi-rater agreement
k/κ	Cohen’s Kappa (inter-rater reliability)
MRI	Magnetic Resonance Imaging
MRE	Magnetic Resonance Enterography
NIHR	National Institute for Health and Care Research
NHS	National Health Service
NPV	Negative Predictive Value
OE	Optical Endoscopy
PCE	Panenteric capsule endoscopy
PEG	Polyethylene Glycol
PPV	Positive Predictive Value
PRISMA	Preferred Reporting Items for Systematic Reviews and Meta-Analyses (referenced in style, not acronym use)
R	R Programming Language (used for analysis)
RAPID	Rapid Access Real-Time Device (Medtronic software)
REC	Research Ethics Committee
S1/S2/S3	Supplementary Tables/Figures (and their annotations, respectively)
SD	Standard Deviation
TOSTs	Two One-Sided Tests (equivalence testing)
TransUNet	Neural Network Architectures for image segmentation
