# Supplementary material for: Inter- and Intraobserver Variability in Bowel Preparation Scoring for Colon Capsule Endoscopy: Impact of AI-Assisted Assessment Feasibility Study
Source: Cancers (Basel). 2025 Aug 29;17(17):2840. doi: 10.3390/cancers17172840 (PMC12427401; doi:10.3390/cancers17172840)
Supplement: Supplementary file 1 [file cancers-17-02840-s001.zip › cancers-3762495-supplementary.pdf]

Supplementary Materials

Table S1. Inclusion and exclusion criteria for NHS England Criteria used in the CESCAIL study [4]

Patient type	Inclusion criteria	Exclusion criteria
All	- Over 18 years of age<br>- Able to provide valid consent	- Difficulty swallowing<br>- Indwelling electromedical device<br>- Diabetes<br>- Past medical history of stricture<br>- Pregnancy<br>- Unfit for bowel preparation
Symptomatic	- Referred from the primary care under the lower gastrointestinal two week wait arm<br>- assessed by a secondary care consultant	- Predominant referral symptom of diarrhoea<br>- F-Hb ≥100 μg/g<br>- Microcytic anaemia as the sole investigation reason
Surveillance	- Due post polypectomy surveillance colonoscopy within a month before, during and after the recruitment period.<br>- More than 5 polyps in last colonoscopy<br>- Presence of one or more advance polyp (≥10mm in size), serrated polyp with dysplasia, adenoma with high-grade dysplasia [5]	- Family history of colorectal cancer<br>- History of colonic polyposis<br>- Hereditary non-polyposis syndrome

Table S2. The CCE Readers List.
To ensure inclusivity and reflect real-world practice, the panel of CCE readers included capsule endoscopy nurses, gastroenterology fellows, consultant gastroenterologists, and a colorectal surgeon.

CCE readers	Affiliation
Chander Shekhar	Institute of Precision Diagnostics & Translational Medicine, University Hospital of Coventry and Warwickshire, Clifford Bridge Rd, Coventry CV2 2DX
Bei Bei Lui	Institute of Precision Diagnostics & Translational Medicine, University Hospital of Coventry and Warwickshire, Clifford Bridge Rd, Coventry CV2 2DX
Alexander Robertson	Department of Digestive Diseases, University Hospitals of Leicester NHS Trust, Leicester, UK.
Ursula Valentiner	Institute of Anatomy and Experimental Morphology, University Medical Center Hamburg-Eppendorf, Hamburg, Germany.
Benedicte Schelde-Olesen	Department of Surgery, Odense University Hospital, 5700 Svendborg, Denmark.
Anirudh Bhandare	Department of Gastroenterology, Royal Oldham Hospital, Northern Care Alliance, Rochdale Road, Oldham, OL1 2JH
Ian Io Lei	Institute of Precision Diagnostics & Translational Medicine, University Hospital of Coventry and Warwickshire, Clifford Bridge Rd, Coventry CV2 2DX
Alice Mapiye	Department of Digestive Diseases, University Hospitals of Leicester NHS Trust, Leicester, UK.
Daniel Gaya	Department of Gastroenterology, University of Glasgow, Glasgow, Scotland.

Table S3. CCE readers and experience		
CCE readers	Estimated Lifetime CCE Reading Volume	Categories
Reader 0	2000	Experienced
Reader 1	700	Experienced
Reader 2	650	Experienced
Reader 3	700	Experienced
Reader 4	500	Experienced
Reader 5	180	Less experienced
Reader 6	150	Less experienced
Reader 7	200	Less experienced
Reader 8	450	Less experienced

Table S4. Summary of Paired TSOT							
Rater	Mean Difference	NHST p-value	TOST p-value	90% CI (TOST)	Equivalence Bounds	TOST Conclusion	NHST Conclusion
Reader 1	-1.455	0.000329	0.288	[-0.5, -0.193]	±0.398	Not Equivalent	Significant
Reader 2	-1.21	1.73E-07	0.923	[-0.55, -0.303]	±0.320	Not Equivalent	Significant
Reader 3	-1.067	5.29E-27	1.0	[-1.202, -0.931]	±0.352	Not Equivalent	Significant
Reader 4	-0.627	3.33E-12	1.0	[-0.752, -0.501]	±0.327	Not Equivalent	Significant
Reader 5	-1.2	3.8E-31	1.0	[-1.345, -1.055]	±0.377	Not Equivalent	Significant
Reader 6	-1.72	9.28E-27	1.0	[-1.892, -1.548]	±0.447	Not Equivalent	Significant
Reader 7	-2.0	3.2E-24	1.0	[-2.221, -1.779]	±0.575	Not Equivalent	Significant
Reader 8	-0.386	8.27E-05	0.435	[-0.541, -0.232]	±0.402	Not Equivalent	Significant

Table S5. Sensitivity Analysis of Manual Reads Using Bootstrapped Fleiss' Kappa			
Observer removed	ICC	95 CI	P value
DRG	0.447645	0.425, 0.476	0.524
IIL	0.456974	0.432,0.482	0.860
BSO	0.450055	0.424, 0.476	0.730
AM	0.461524	0.437, 0.488	0.638
AR	0.45249	0.427, 0.479	0.836
BBL	0.461657	0.435, 0.490	0.642
CS	0.458583	0.436, 0.486	0.802
AB	0.46614	0.442, 0.494	0.354
UV	0.440514	0.418, 0.468	0.270

Table S6. Sensitivity Analysis of Manual Reads Using Bootstrapped ICC			
Observer removed	ICC	95 CI	P value
DRG	0.916263	0.903, 0.928	0.774
IIL	0.915513	0.902,0.928	0.764
BSO	0.909894	0.896,0.923	0.594
AM	0.910694	0.897,0.923	0.674
AR	0.914488	0.901, 0.927	0.882
BBL	0.914878	0.902, 0.927	0.852
CS	0.91653	0.904, 0.928	0.686
AB	0.917827	0.905, 0.930	0.602
UV	0.441	0.898, 0.924	0.792

Table S7. Sensitivity Analysis of AI-assisted Reads Using Bootstrapped Fleiss' Kappa			
Observer removed	ICC	95 CI	P value
DRG	0.121	0.094, 0.159	0.538
IIL	0.135	0.104, 0.170	0.854
BSO	0.101	0.078, 0.143	0.066
AM	0.094	0.069, 0.133	0.016*
AR	0.095	0.065, 0.129	0.022*
BBL	0.176	0.144, 0.217	0.022*
CS	0.184	0.153, 0.232	0.010*
AB	0.151	0.121, 0.185	0.290

Table S8. Sensitivity Analysis of AI-assisted Reads Using Bootstrapped ICC			
Observer removed	ICC	95 CI	P value
DRG	0.596	0.522, 0.696	0.868
IIL	0.584	0.503, 0.687	0.944
BSO	0.565	0.479, 0.660	0.620
AM	0.562	0.474, 0.669	0.588
AR	0.564	0.476, 0.655	0.624
BBL	0.608	0.540, 0.696	0.638
CS	0.628	0.557, 0.707	0.296
AB	0.607	0.531, 0.695	0.648

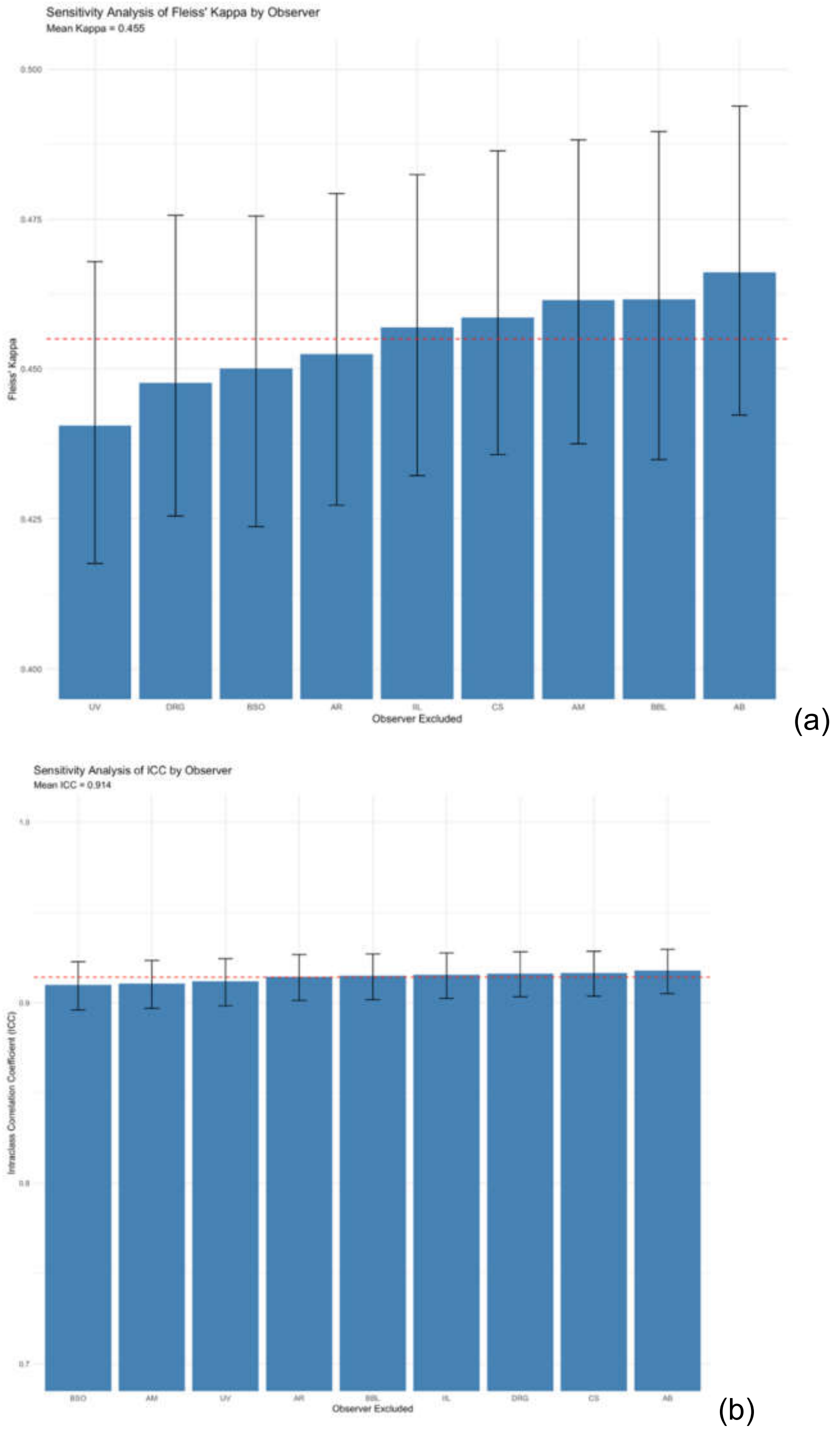

Figure S1. Sensitivity analysis using a leave-one-observer-out approach: (a) Fleiss' Kappa for manual reads; (b) Intraclass Correlation Coefficient (ICC) for manual reads.

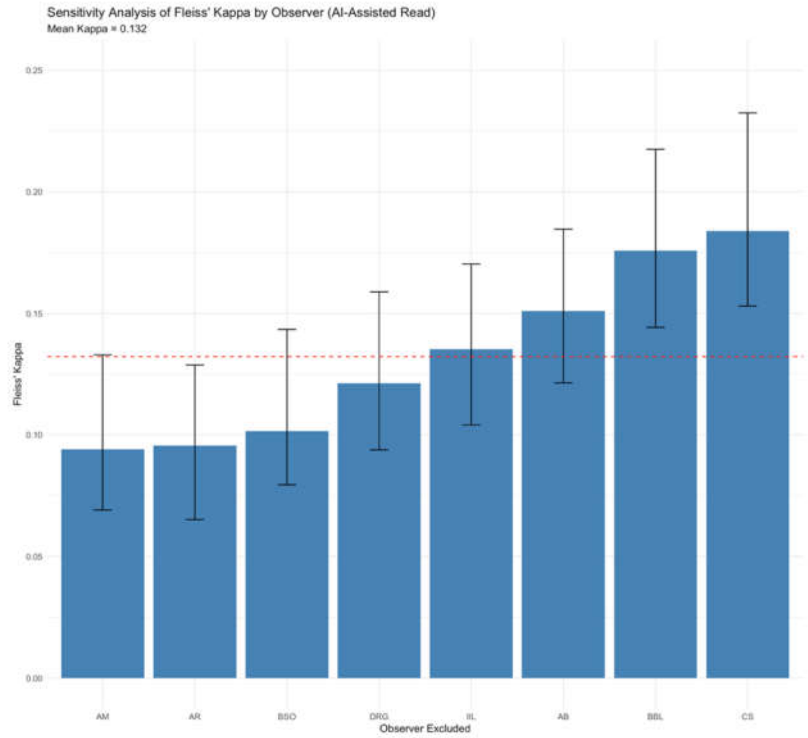

(a)

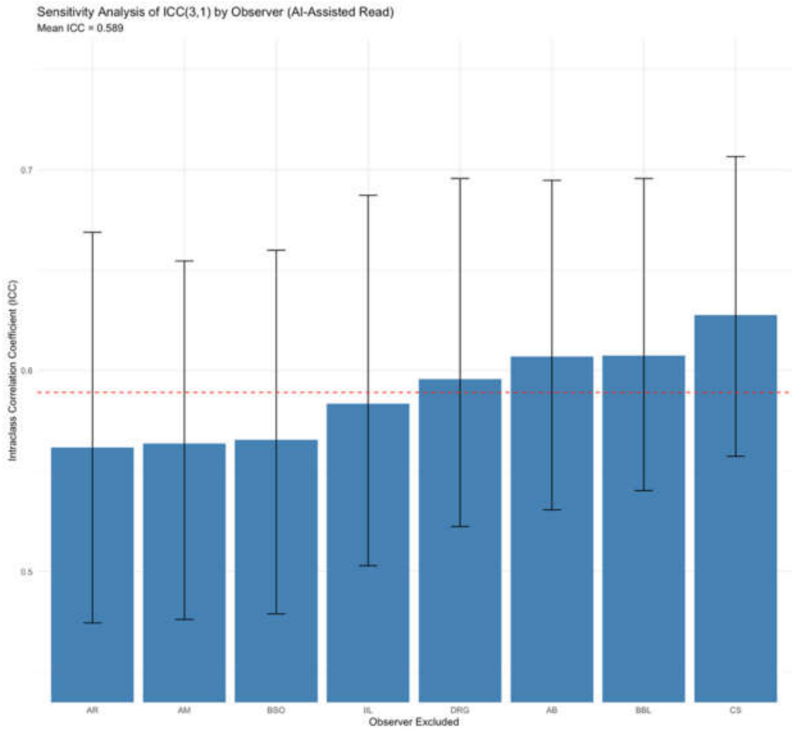

(b)

Figure S2. Sensitivity analysis using a leave-one-observer-out approach: (a) Fleiss' Kappa for AI-assisted reads; (b) Intraclass Correlation Coefficient (ICC) for AI-assisted reads.

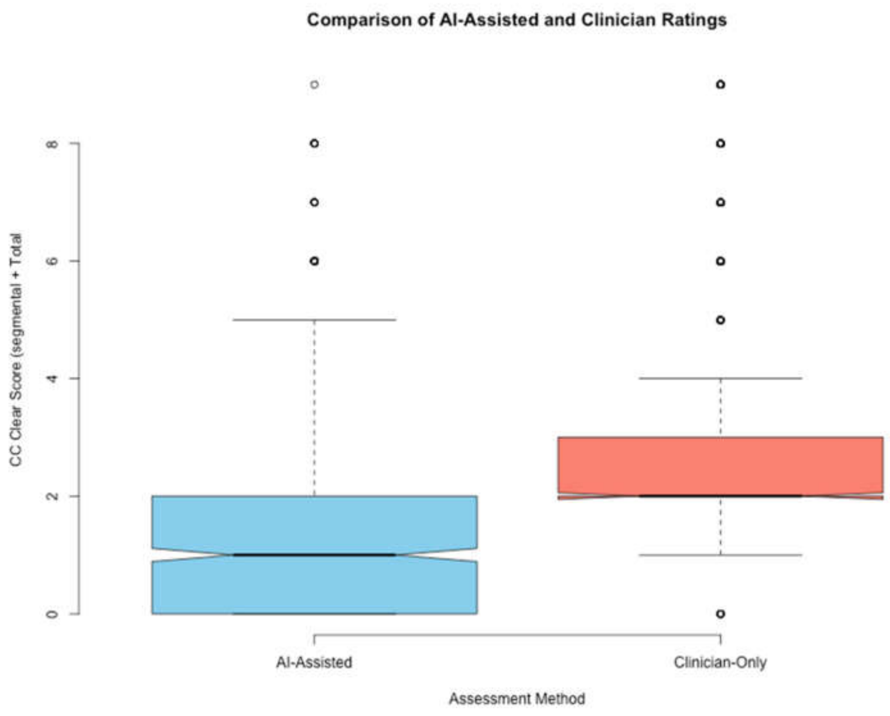

Figure S3. Box plot to compare mean score in the AI-assisted arm against the standard arm using CC Clear score.
